# Polycystin‐1 affects cancer cell behaviour and interacts with mTOR and Jak signalling pathways in cancer cell lines

**DOI:** 10.1111/jcmm.14506

**Published:** 2019-06-28

**Authors:** Kostas A. Papavassiliou, Ilianna Zoi, Antonios N. Gargalionis, Michael Koutsilieris

**Affiliations:** ^1^ Department of Physiology, Medical School National and Kapodistrian University of Athens Athens Greece; ^2^ Department of Biological Chemistry, Medical School National and Kapodistrian University of Athens Athens Greece; ^3^ Department of Biopathology Aeginition Hospital, National and Kapodistrian University of Athens Athens Greece

**Keywords:** cancer, cell migration, cell proliferation, Jak signalling, mTOR signalling, PC1, polycystins

## Abstract

Polycystic Kidney Disease (PKD), which is attributable to mutations in the *PKD1* and *PKD2* genes encoding polycystin‐1 (PC1) and polycystin‐2 (PC2) respectively, shares common cellular defects with cancer, such as uncontrolled cell proliferation, abnormal differentiation and increased apoptosis. Interestingly, PC1 regulates many signalling pathways including Jak/STAT, mTOR, Wnt, AP‐1 and calcineurin‐NFAT which are also used by cancer cells for sending signals that will allow them to acquire and maintain malignant phenotypes. Nevertheless, the molecular relationship between polycystins and cancer is unknown. In this study, we investigated the role of PC1 in cancer biology using glioblastoma (GOS3), prostate (PC3), breast (MCF7), lung (A549) and colorectal (HT29) cancer cell lines. Our in vitro results propose that PC1 promotes cell migration in GOS3 cells and suppresses cell migration in A549 cells. In addition, PC1 enhances cell proliferation in GOS3 cells but inhibits it in MCF7, A549 and HT29 cells. We also found that PC1 up‐regulates mTOR signalling and down‐regulates Jak signalling in GOS3 cells, while it up‐regulates mTOR signalling in PC3 and HT29 cells. Together, our study suggests that PC1 modulates cell proliferation and migration and interacts with mTOR and Jak signalling pathways in different cancer cell lines. Understanding the molecular details of how polycystins are associated with cancer may lead to the identification of new players in this devastating disease.

## 1 INTRODUCTION

Polycystin‐1 (PC1) and polycystin‐2 (PC2) are known for their major role in autosomal dominant polycystic kidney disease (ADPKD) where mutations in the genes encoding both polycystins result in the generation of fluid‐filled renal cysts as well as cysts in other epithelial organs including the liver and the pancreas.^1^ Almost 85% of the ADPKD cases result from mutations in the *PKD1* gene on chromosome 16 that encodes PC1,^2^ whereas mutations in the *PKD2* gene on chromosome 4 encoding PC2, are responsible for the remaining 15% of the cases.^3^, ^4^ PC1 is a large transmembrane protein and consists of a long extracellular domain, 11 transmembrane domains and a short intracellular domain 5, 6 that regulates various signalling pathways^7^ including Wnt signalling pathway,^8^ AP‐1 transcription factor complex signalling,^9^, ^10^ STAT6 signalling,^11^ and mTOR signalling.^12^, ^13^, ^14^, ^15^ PC1 has been localized at cell‐cell contacts where it modulates cell adhesion^16^, ^17^ and to cell‐matrix contacts.^18^ PC1 has also been located at the primary cilium of kidney cells, where it is thought to act as a mechanosensitive receptor that transduces mechanical stimuli (fluid flow) into intracellular biochemical signals.^19^, ^20^, ^21^ PC2 is a smaller transmembrane protein that contains six transmembrane domains, with intracellular C‐ and N‐termini.^3^, ^22^ PC2 belongs to the transient receptor potential family of calcium channels that regulate intracellular calcium and affects various cellular features such as cell proliferation, differentiation and planar cell polarity.^23^, ^24^, ^25^ Accumulating evidence suggests that both polycystins act as conductors to tune the overall mechanosensitivity of cells.^26^

The function of polycystins has mainly been explored in the context of PKD where mutations in the polycystins PC1 and PC2 give rise to a complex cell phenotype, characterized by increased cell proliferation and apoptosis, de‐differentiation, disturbed planar cell polarity, extracellular matrix alterations and abnormal fluid secretion.^27^ In cancer, however, the function of polycystins is unknown. A comparison between cancer and PKD reveals that both diseases exhibit a deregulation in many important cellular features, such as proliferation, differentiation and apoptosis.^27^, ^28^ Surprisingly, ADPKD cells activate some of the same signalling pathways that are utilized by cancer cells in order to promote their malignant cell behaviour. For example, the mTOR pathway is a critical pathway that is deregulated in both cancer and PKD. mTOR signalling is up‐regulated in a wide variety of cancers and is regarded as one of the most frequently altered cascades in this heterogeneous disease.^29^, ^30^, ^31^ mTOR signalling is increased in mouse models of PKD and human ADPKD, while mTOR inhibitors, such as sirolimus and everolimus, slow disease progression in PKD animal models.^12^, ^32^, ^33^, ^34^ The Jak/STAT pathway is also deregulated in both cancer and PKD. Jak/STAT signalling is activated in haematological malignancies, particularly in myeloproliferative neoplasms and solid tumours.^35^, ^36^, ^37^ In PKD, Jak/STAT signalling activity is abnormally activated and promotes cystic growth.^38^, ^39^, ^40^, ^41^, ^42^

Despite these similarities between cancer and PKD, up to date, there is only one study on the function of polycystins in cancer. Analysing colorectal cancer (CRC) cell lines (HCT116, HT29 and SW480), HT29 tumour xenografts and cancer tissue samples from CRC patients, Gargalionis et al provided evidence of a role for polycystins in CRC aggressiveness.^43^ In the present study, our goal was to examine the in vitro role of PC1 in cancer using cancer cell lines derived from five different types of human cancer (brain—GOS3, lung—A549, prostate—PC3, colon—HT29, breast—MCF7). We found that PC1 modulates the proliferation and migration of cancer cells. We also found that PC1 interacts with mTOR and Jak signalling and affects their activity in cancer cells. Importantly, our study represents the first steps in understanding the function of polycystins in cancer biology.

## 2 MATERIALS AND METHODS

### 2.1 Cell cultures

MCF7, PC3, A549, HT29 and CACO2 cell lines were cultured in DMEM containing L‐glutamine, 4.5 g/L D‐glucose and pyrophosphate sodium supplemented with 10% foetal bovine serum (FBS) and 1% penicillin‐streptomycin (10 000 U/mL penicillin‐10 000 mg/mL streptomycin). GOS3 cancer cells were cultured in RPMI 1640 medium GlutaMAX supplemented with 10% FBS, 1% penicillin‐streptomycin (10 000 U/mL penicillin‐10 000 µg/mL streptomycin). CHLA‐259 cells were grown in a base medium of Iscove's Modified Dulbecco's Medium supplemented with 20% FBS, 4 mmol/L L‐Glutamine, 1× ITS (5 µg/mL insulin, 5 µg/mL transferrin, 5 ng/mL selenous acid). HBEC3‐KT cells were cultured in Airway Epithelial Cell Basal Medium (ATCC PCS‐300‐030) supplemented with Bronchial Epithelial Cell Growth Kit (ATCC PCS‐300‐040). MCF10A cells were cultured in DMEM/F12 plus 5% horse serum supplemented with penicillin, streptomycin, L‐glutamine, 20 ng/mL epidermal growth factor, 0.5 μg/mL hydrocortisone, 10 μg/mL insulin and 100 ng/mL cholera toxin. HPrEc cells were cultured in Prostate Epithelial Cell Basal Medium (ATCC PCS‐440‐030) supplemented with Prostate Epithelial Cell Growth Kit (ATCC PCS‐440‐040). All cell cultures were maintained at 37°C in a humidified atmosphere containing 5% CO2‐95% air.

### 2.2 PC1 knockdown

MCF7, PC3, A549, HT29 and GOS3 cancer cells were transfected with Dharmacon's chemically synthesized siRNA SMARTpools [human PC‐1, L‐007666‐00‐0005, ON‐TARGETplus Human PKD1 (5310) siRNA—SMARTpool, 5 nmol] and non‐targeting siRNA for control cells (D‐001210‐01‐05, siGENOME Non‐Targeting siRNA #1, 5 nmol), in dilution 1:20 in 1× siRNA buffer, using DharmaFECT 2 Transfection Reagent, 0.2 mL (Dharmacon) in dilution 1:50 in DMEM (Gibco, Thermo Fisher Scientific). Cell starvation was performed for 6 hours before transfection in order to achieve proper cell cycle synchronization.

### 2.3 Antibodies

The following primary antibodies were used for Western blot analysis: Polycystin‐2 (sc‐10376 Santa Cruz Biotechnology), p70‐S6K (sc‐230 Santa Cruz Biotechnology), phospho‐p70‐S6K (sc‐8416 Santa Cruz Biotechnology), phospho‐mTOR (5536 CST), phospho‐4E‐BP1 (2855 CST), PTEN (9559 CST), Akt (9272 CST), phospho‐Akt (9271 CST), actin (MAB1501 Millipore), polycystin‐1 CT2741 (kindly provided by the Baltimore Polycystic Kidney Disease Research and Clinical Core Center), mTOR (701483 Thermo Fisher Scientific), 4EBP1 (AHO1382 Thermo Fisher Scientific), JAK2 (ab37226 Abcam), phospho‐JAK2 (ab32101 Abcam). The following secondary antibodies were used: goat anti‐mouse IgG HRP‐conjugate (AP124P Millipore), goat anti‐rabbit IgG HRP‐conjugate (AP132P Millipore), donkey anti‐goat IgG HRP‐conjugate (A00178 GenScript). The IgPKD1 inhibitory antibody was a generous gift from Dr O. Ibraghimov‐Beskrovnaya and H. Husson (Genzyme Co., Boston).

### 2.4 Semi‐quantitative PCR and quantitative real‐time PCR

Total RNA was extracted from cultured cells using RNeasy Mini Kit (Qiagen, Hilden, Germany) according to the manufacturer's instructions. PrimeScript RT reagent kit‐Perfect Real Time (Takara Bio, Japan) for RT‐PCR was used for cDNA synthesis according to the manufacturer's protocol.

For semi‐quantitative PCR, the produced cDNA was amplified with specific primer pairs for PC1‐encoding *Pkd1* (annealing 58°C, forward CGCCGCTTCACTAGCTTCGAC; reverse ACGCTCCAGAGGGAGTCCAC) and PC2‐encoding *Pkd2* (annealing 53°C, forward GCGAGGTCTCTGGGGAAC; reverse TACACATGGAGCTCATCATGC) genes (35 cycles) as well as with actin gene primer pairs (28 cycles) using KAPA2G Fast Multiplex PCR Kit (KK5801, Kapa Biosystems). PCR‐amplified fragments were analysed after their separation in agarose gels using image analysis software (Image J; La Jolla, CA) and normalized to actin gene levels.

Quantitative real‐time PCR was performed with an iCycler real‐time instrument (Bio‐Rad Laboratories, Hercules, CA) and the RT‐PCR product was amplified using the iQ SYBR Green Supermix (Bio‐Rad). Primer pairs were used for the *Pkd1* (annealing 61°C, forward CAAGACACCCACATGGAAACG; reverse CGCCAGCGTCTCTGTCTTCT) gene (40 cycles) normalized to actin gene levels (annealing 62°C).

### 2.5 Western Blot analysis

Proteins were resolved by electrophoresis in SDS‐polyacrylamide gels with varying densities (6% for PC1; 8% for mTOR and p‐mTOR; 10% for PC2, Jak2 and p‐Jak2; 12% for p70S6K, p‐p70S6K, Akt, p‐Akt and PTEN; 15% for 4EBP1 and p‐4EBP1) and transferred to a nitrocellulose membrane (Porablot NCP, Macherey‐Nagel, Duren, Germany). Membranes were incubated overnight at 4°C with the primary antibodies (dilutions were 1:250 for antibodies against PC1, PC2, mTOR, 4EBP1, p70S6K, p‐p70S6K; 1:500 for Jak2 and p‐Jak2; 1:1000 for p‐mTOR, Akt, p‐Akt, PTEN, p‐4EBP1, actin in PBST containing 1% non‐fat milk). Detection of the immunoreactive bands was performed with the LumiSensor Chemiluminescent HRP Substrate kit (GenScript, NJ). Relative protein amounts were evaluated by a densitometric analysis using Image J software and normalized to the corresponding actin levels.

### 2.6 Cell Proliferation Assay

Cells were seeded in a 96‐well plate at a density of 10^3^‐10^5^ cells/well in 100 μL of culture medium with IgPKD1 (1:50 and 1:100 dilutions) or non‐immune rabbit serum. Cells were cultured in a CO_2_ incubator at 37°C for 24 and 48 hours. Ten microlitres of the prepared XTT Mixture (XTT Cell Proliferation Assay Kit, 10010200; Cayman Chemical, USA) was added to each well and mixed gently. The cells were incubated for 4 hours at 37°C in a CO_2_ incubator. The absorbance of each sample was measured using a microplate reader at a wavelength of 450 nm. Cells were synchronized with serum starvation for 6 hours.

### 2.7 Cell migration assay

HT29, MCF7, PC3, A549 and GOS3 cells were cultured in 12‐well cell plates until confluent and synchronized by serum starvation for 6 hours. The cellular layer was etched with a 200 μL sterile pipette tip. Cells were incubated with IgPKD1 or non‐immune rabbit serum. Each location was photographed in a computer‐connected microscope (×10 magnification) at zero hour and after 24 hour incubation. Images were analysed using TScratch software. The results were expressed as percentages of the incised and the cell‐coated region.

### 2.8 Statistical and image analysis

All experiments were performed at least three times. Data are presented as mean ± SD and were analysed by one‐way ANOVA. GraphPad Prism 6 software was employed for these statistical analyses. All statistical tests were two‐sided. *P* < 0.05 was considered statistically significant.

## 3 RESULTS

### 3.1 Endogenous mRNA and protein expression of PC1 and PC2 in cell lines

**Figure 1:**
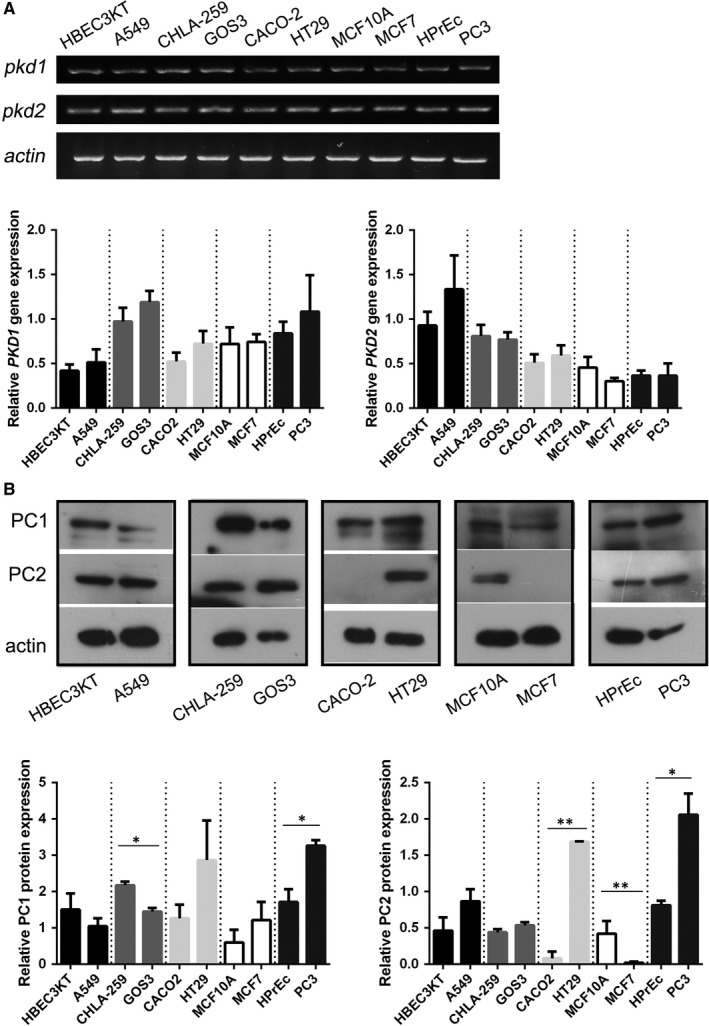
Endogenous *Pkd1* and *Pkd2* mRNA and PC1 and PC2 protein expression in cell lines. (A) Semi‐quantitative PCR analysis showing *Pkd1* and *Pkd2* mRNA levels in HBEC3KT, A549, CHLA‐259, GOS3, CACO2, HT29, MCF10A, MCF7, HPrEc, PC3 cells. Actin was used as a mRNA loading control. Bars represent means ± SD. (B) Western blot analysis of PC1 and PC2 protein levels in HBEC3KT, A549, CHLA‐259, GOS3, CACO2, HT29, MCF10A, MCF7, HPrEc, PC3 cells. Actin was used as a protein loading control. Bars represent means ± SD. *P < 0.05, **P < 0.01 versus respective normal cell line (CHLA‐259 is the respective normal cell line of GOS3, HPrEc is the respective normal cell line of PC3, CACO2 is the respective normal cell line of HT29, MCF10A is the respective normal cell line of MCF7)

PC1 and PC2 proteins have only been detected in SW480 CRC cells,^43^ therefore we firstly sought to determine the endogenous mRNA and protein expression levels of the two polycystins in MCF7, PC3, A549, HT29 and GOS3 cancer cell lines. We detected both mRNA (Figure 1A) and protein (Figure 1B) levels of PC1 and PC2 in all cell lines apart from PC2 protein in MCF7 cells. There were discrepancies between *Pkd1* mRNA levels and PC1 protein levels in some cancer cell lines, as well as discrepancies between *Pkd2* mRNA levels and PC2 protein levels. For example, in MCF7 cells the *Pkd2* gene expression is increased but the PC2 protein expression is negligible. These differences may be due to post‐transcriptional and post‐translational regulatory mechanisms. In addition, we compared the mRNA and protein levels in the cancer cell lines to the levels in normal cell lines from the same embryonic origin. Our results show that PC1 protein levels were higher in prostate cancer cells (PC3) compared to normal cells (HPrEc) and lower in glioblastoma cells (GOS3) compared to normal brain cells (CHLA‐259). PC2 protein levels were found to be higher in CRC (HT29) and prostate cancer (PC3) cells compared to normal cells (CACO2 and HPrEc respectively), while they were lower in breast cancer cells (MCF7) compared to normal breast cells (MCF10A) (Figure 1B). There were no differences observed in the mRNA levels of PC1 and PC2 between cancer and normal cell lines.

### 3.2 Effect of antibody‐mediated PC1 inhibition on cell migration and proliferation in cancer cell lines

**Figure 2:**
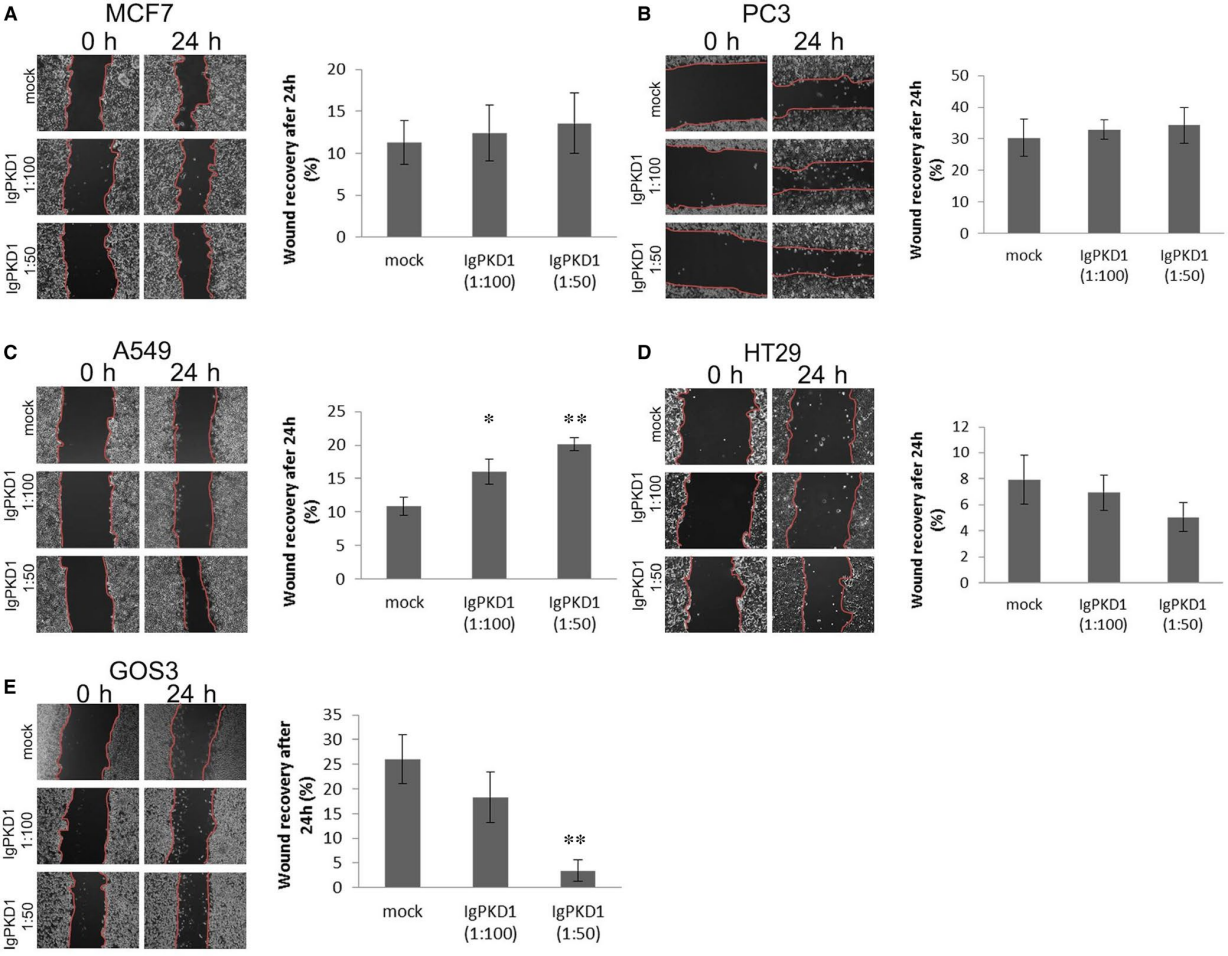
Effect of PC1 inhibition on cancer cell migration. (A‐E) Cell migration assay in MCF7, PC3, A549, HT29 and GOS3 cells. IgPKD1 is the inhibitory antibody against PC1. Mock represents cells that have been incubated with non‐immune rabbit serum (without the IgPKD1 antibody). The images were analysed using TScratch software. Bars represent mean areas ± SD. *P < 0.05, **P < 0.01 versus mock

**Figure 3:**
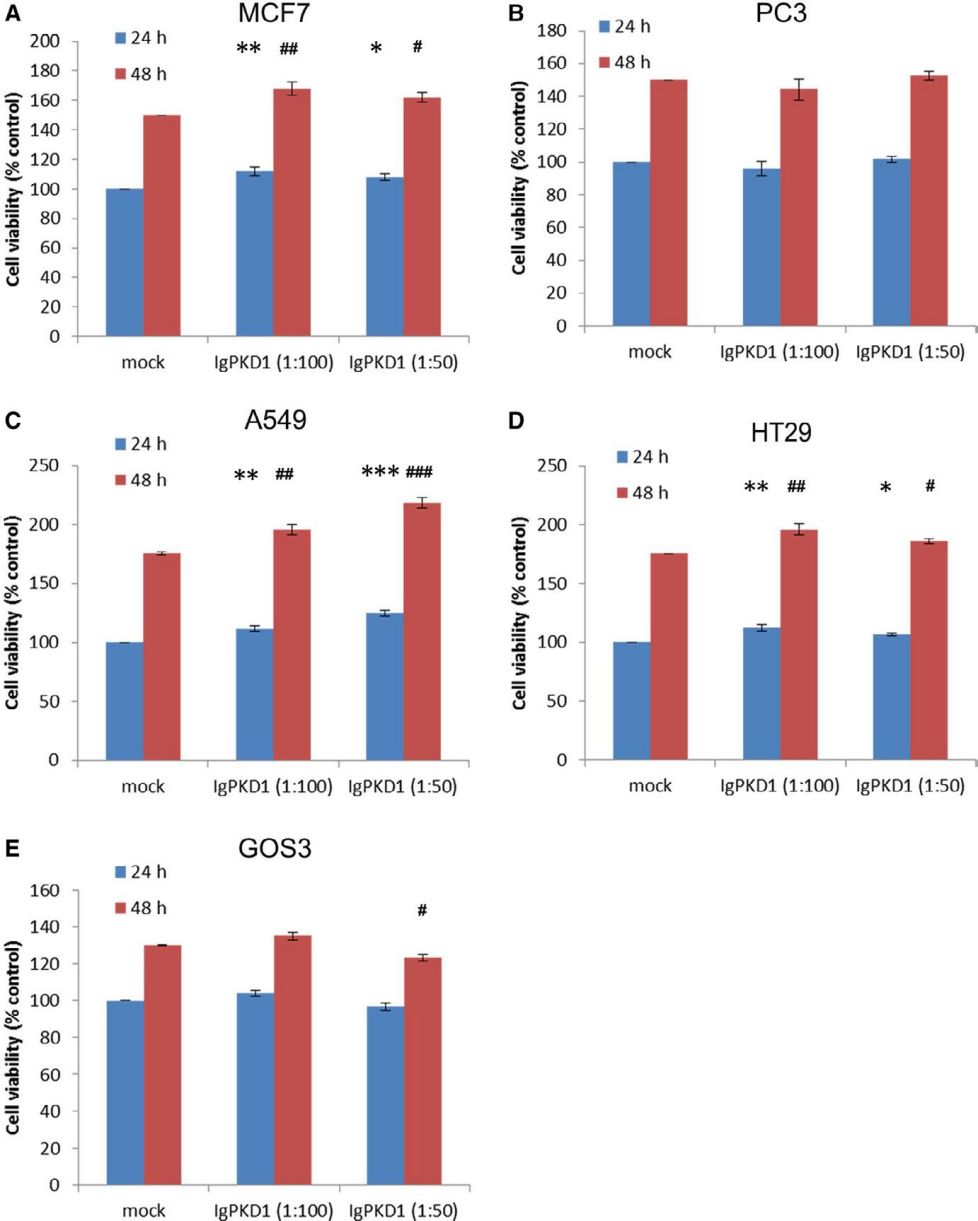
Effect of PC1 inhibition on cancer cell proliferation. (A‐E) Cell proliferation assay in MCF7, PC3, A549, HT29 and GOS3 cells. IgPKD1 is the inhibitory antibody against PC1. Mock represents cells that have been incubated with non‐immune rabbit serum (without the IgPKD1 antibody). Each bar represents mean ± SD. *P < 0.05, **P < 0.01, ***P < 0.001 versus mock at 24 hours. ^#^ *P* < 0.05, ^##^ *P* < 0.01, ^###^ *P* < 0.001 versus mock at 48 h

Next, we wanted to explore if PC1 affects cancer cell behaviour. Thus, we decided to investigate whether PC1 affects cell migration and proliferation in cancer cell lines by incubating them with a blocking antibody, IgPKD1, raised against the Ig‐like domains of extracellular PC1.^16^ Even though the function of PC1 remains obscure, and hence, there is still no specific assay to show that PC1 is inhibited, the IgPKD1 antibody is a valid method of inhibiting PC1. IgPKD1 has been used to block PC1 in murine, canine and human kidney epithelial cells,^16^, ^17^, ^44^ bone cells,^45^, ^46^ CRC cells and xenografts^43^ and endothelial cells.^47^ We found that in A549 cells, IgPKD1 treatment led to increased cell migration with the greatest effect observed at a 1:50 dilution of the IgPKD1 antibody (Figure 2C). Conversely, in GOS3 cells, blocking PC1 resulted in decreased cell migration with the greatest effect observed at a 1:50 dilution of IgPKD1 (Figure 2E). These results suggest that PC1 function in vitro is cancer cell type‐specific, promoting cell migration in GOS3 cells and suppressing cell migration in A549 cells. In terms of cell proliferation, our results show that MCF7, A549 and HT29 cells exhibited increased cell viability at both 24 and 48 hours after PC1 inhibition (Figure 3A,3C,3D). However, in GOS3 cells, cell proliferation decreased at 48 hours after PC1 inhibition (1:50 dilution of the IgPKD1 antibody) (Figure 3E). PC3 cells showed no significant effect on cell proliferation (Figure 3B). These data indicate that PC1 enhances cell proliferation in GOS3 cells but hinders it in MCF7, A549 and HT29 cells.

### 3.3 Effect of Pkd1 silencing on mTOR pathway in cancer cell lines

**Figure 4:**
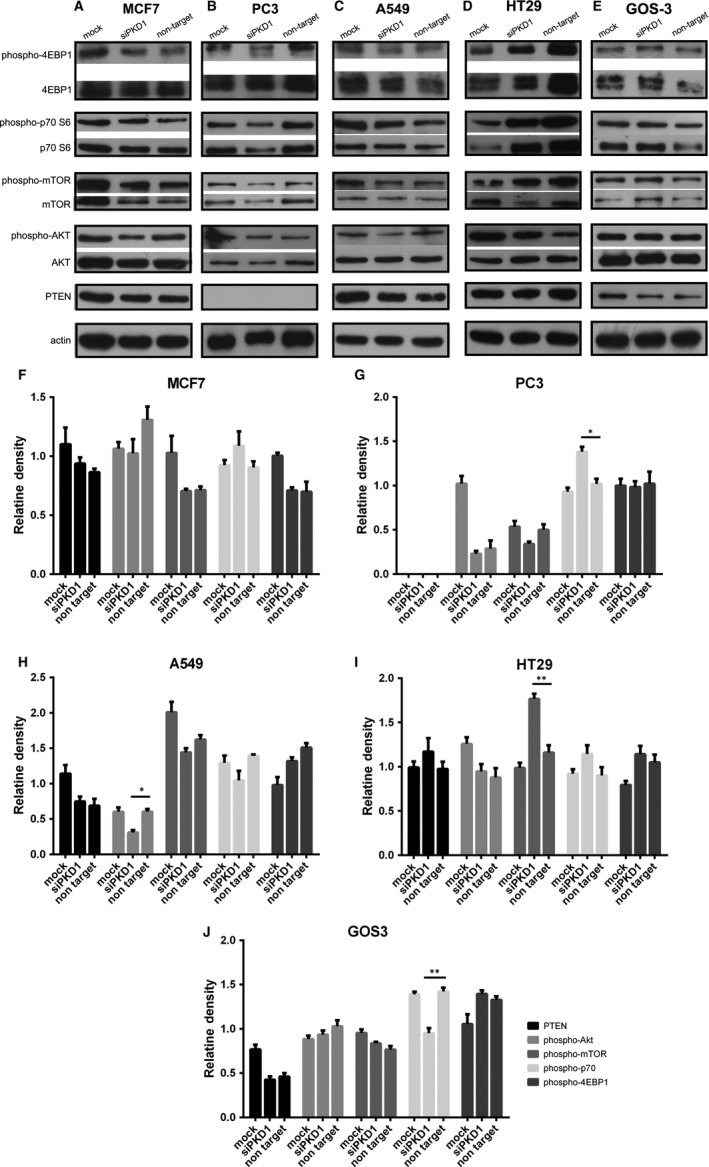
Effect of Pkd1 silencing on the mTOR pathway in cancer cell lines. (A‐E) Western blot analysis showing the effect of Pkd1 silencing on the phosphorylation of mTOR pathway components in MCF7, PC3, A549, HT29 and GOS3 cells. (F‐J) Quantitative data showing the effect of Pkd1 silencing on the phosphorylation of mTOR pathway components in MCF7, PC3, A549, HT29 and GOS3 cells. Bars represent means ± SD. *P < 0.05, **P < 0.01 versus non‐target. siPKD1 represents cancer cells transfected with siRNA targeting the *Pkd1* mRNA; non‐target represents cancer cells transfected with a non‐targeting siRNA; mock represents cancer cells transfected with only transfection reagents (without siRNA)

Subsequently, we sought to explore the effect of PC1 on mTOR signalling in our cancer cell lines when PC1 protein expression is knocked down by siRNA. The knockdown efficiency of the *Pkd1* siRNA was confirmed by quantitative real‐time PCR (qRT‐PCR) (Figure S1). Our results revealed that p70S6K phosphorylation was increased in PC3 cells treated with siRNA targeting the *Pkd1* mRNA (siPKD1) (Figure 4B,G), while it was decreased in GOS3 cells treated with siRNA targeting the *Pkd1* mRNA (siPKD1) (Figure 4E,J). mTOR phosphorylation was increased in HT29 cells treated with siRNA targeting the *Pkd1* mRNA (siPKD1) (Figure 4D,I). Akt phosphorylation was decreased in A549 cells treated with siRNA targeting the *Pkd1* mRNA (siPKD1) (Figure 4C,H). It should be mentioned that PC3 cells are PTEN‐deficient. PC1 knockdown resulted in significantly affecting the phosphorylation of only one mTOR pathway component in most cancer cell lines; therefore, based on our data, PC1 appears to down‐regulate mTOR signalling in PC3 and HT29 cells, while it up‐regulates mTOR signalling in GOS3 and A549 cells. All these results suggest that PC1 interacts in vitro with the mTOR pathway in cancer cells.

### 3.4 Effect of antibody‐mediated PC1 inhibition on mTOR pathway in cancer cell lines

**Figure 5:**
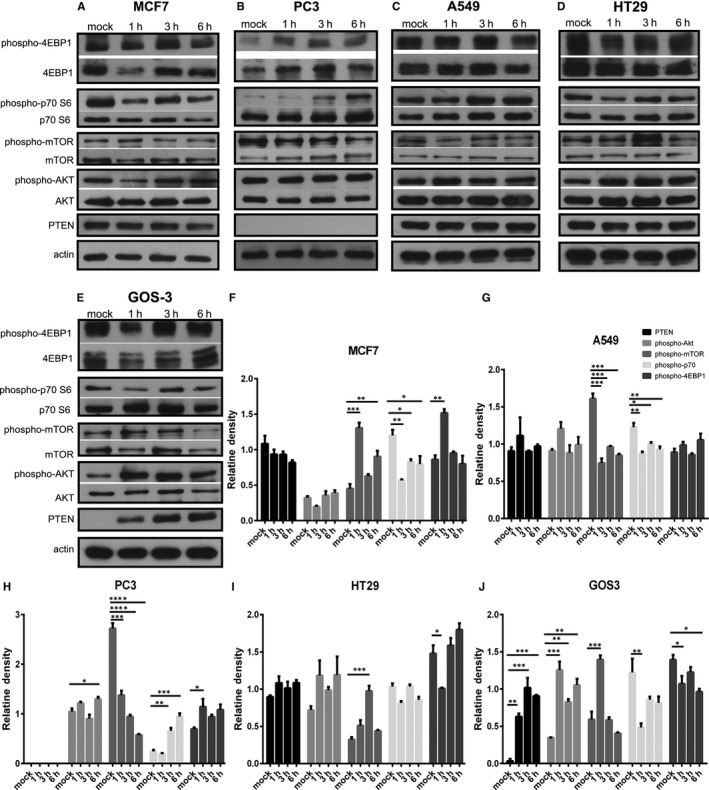
Effect of PC1 inhibition on the mTOR pathway in cancer cell lines. (A‐E) Western blot analysis showing the effect of IgPKD1 on the phosphorylation of mTOR pathway components in MCF7, PC3, A549, HT29 and GOS3 cells. (F‐J) Quantitative data showing the effect of IgPKD1 on the phosphorylation of mTOR pathway components in MCF7, PC3, A549, HT29 and GOS3 cells. Bars represent means ± SD. *P < 0.05, **P < 0.01, ***P < 0.001, ****P < 0.0001 versus mock. Mock represents cells that have been incubated for 3 h with non‐immune rabbit serum; 1 h, 3 h, and 6 h represent time points of cell harvesting after 3 h incubation of cancer cells with the IgPKD1 antibody

In a similar fashion to the above experiments, we investigated the effect of PC1 on mTOR signalling in our cancer cell lines, but this time we inhibited PC1 with the blocking antibody IgPKD1. This was done by incubating all cancer cells with IgPKD1 for 3 hours, followed by collection and analysis of protein extracts at different time points (1, 3, and 6 hours) so as to investigate if the effect of PC1 on mTOR signalling is time‐dependent as well. We show that phosphorylation of mTOR and 4EBP1 increased in MCF7 cells treated with the IgPKD1 antibody. On the other hand, phosphorylation of p70S6K decreased in MCF7 cells treated with the IgPKD1 (Figure 5A,F). In A549 cells, IgPKD1 treatment decreased the phosphorylation of mTOR and p70S6K (Figure 5C,G). In PC3 cells, the phosphorylation of mTOR decreased after IgPKD1 treatment, while the phosphorylation of Akt and p70S6K increased in IgPKD1‐treated cells (Figure 5B,H). In HT29 cells, treatment with IgPKD1 increased mTOR phosphorylation, while it reduced the 4EBP1 phosphorylation (Figure 5D,I). Finally, in GOS3 cells, mTOR and Akt phosphorylation increased in IgPKD1‐treated cells, while the phosphorylation of 4EBP1 and p70S6K decreased in IgPKD1‐treated cells. Total PTEN in GOS3 cells increased in IgPKD1‐treated cells (Figure 5E,J). According to these results, we were not able to clearly identify whether mTOR signalling is up‐ or down‐regulated in each cancer cell line; of the mTOR pathway‐related proteins that we analysed in individual cell lines, some demonstrated increased phosphorylation while others showed decreased phosphorylation. We could probably state that mTOR signalling is up‐regulated in A549 cells, as there is an increase in both mTOR and p70S6K phosphorylation; however, these two proteins were the only ones to show a significant change in their phosphorylation after PC1 inhibition. Our difficulty in determining mTOR pathway activity after PC1 inhibition may be due to the complexity of its regulation which includes activating or inhibitory inputs from several other pathways. Furthermore, our results display a time‐dependent in vitro effect of PC1 on mTOR signalling in cancer cells. For example, in PC3 cells, the phosphorylation of mTOR is gradually decreased over time (1, 3 and 6 hours) in IgPKD1‐treated cells (Figure 5B,H). Taken together, our results further support that PC1 interacts with the mTOR pathway in cancer cells.

### 3.5 Effect of Pkd1 silencing on Jak pathway in cancer cell lines

**Figure 6:**
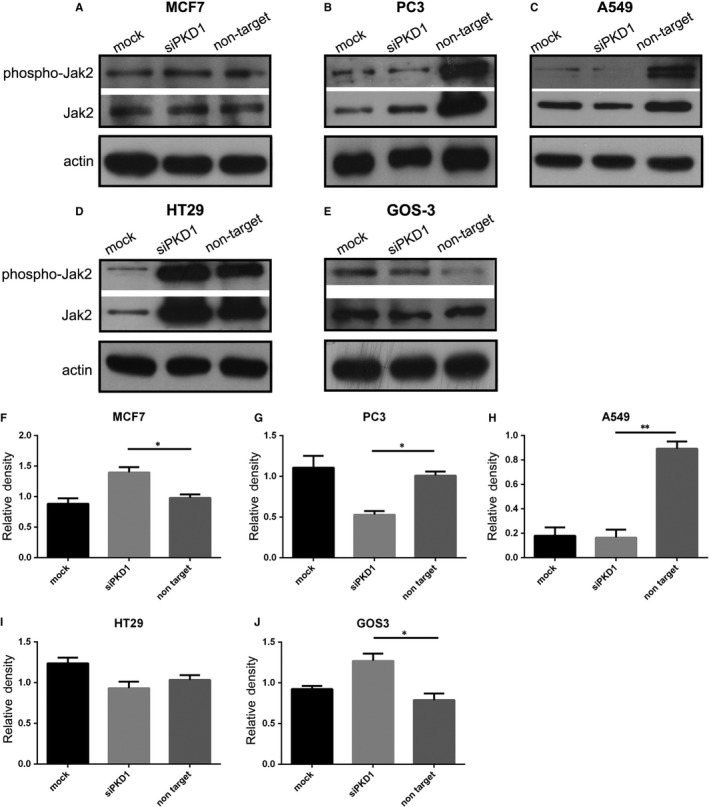
Effect of Pkd1 silencing on the Jak pathway in cancer cell lines. (A‐E) Western blot analysis showing the effect of Pkd1 silencing on the phosphorylation of Jak2 in MCF7, PC3, A549, HT29 and GOS3 cells. (F‐J) Quantitative data showing the effect of Pkd1 silencing on the phosphorylation of Jak2 in MCF7, PC3, A549, HT29 and GOS3 cells. Bars represent means ± SD. *P < 0.05, **P < 0.01 versus non‐target. siPKD1 represents cancer cells transfected with siRNA targeting the *Pkd1* mRNA; non‐target represents cancer cells transfected with a non‐targeting siRNA; mock represents cancer cells transfected with only transfection reagents (without siRNA)

To determine the effect of PC1 on the Jak pathway in our cancer cells, we silenced PC1 protein expression through siRNA. The knockdown efficiency of the *Pkd1* siRNA was confirmed by qRT‐PCR (Figure S1). Our data demonstrate that the phosphorylation of Jak2 is increased in MCF7 (Figure 6A,F) and GOS3 (Figure 6E,J) cells treated with siRNA targeting the *Pkd1* mRNA (siPKD1) compared to MCF7 and GOS3 cells treated with non‐targeting siRNA (non‐target), while it is decreased in PC3 (Figure 6B,G) and A549 (Figure 6C,H) cells treated with siRNA targeting the *Pkd1* mRNA (siPKD1) compared to PC3 and A549 cells treated with non‐targeting siRNA (non‐target). According to this evidence, PC1 down‐regulates Jak signalling in MCF7 and GOS3 cells, whereas it up‐regulates Jak signalling in PC3 and A549 cells. These results suggest that PC1 interacts with the Jak pathway in cancer cells.

### 3.6 Effect of antibody‐mediated PC1 inhibition on Jak pathway in cancer cell lines

**Figure 7:**
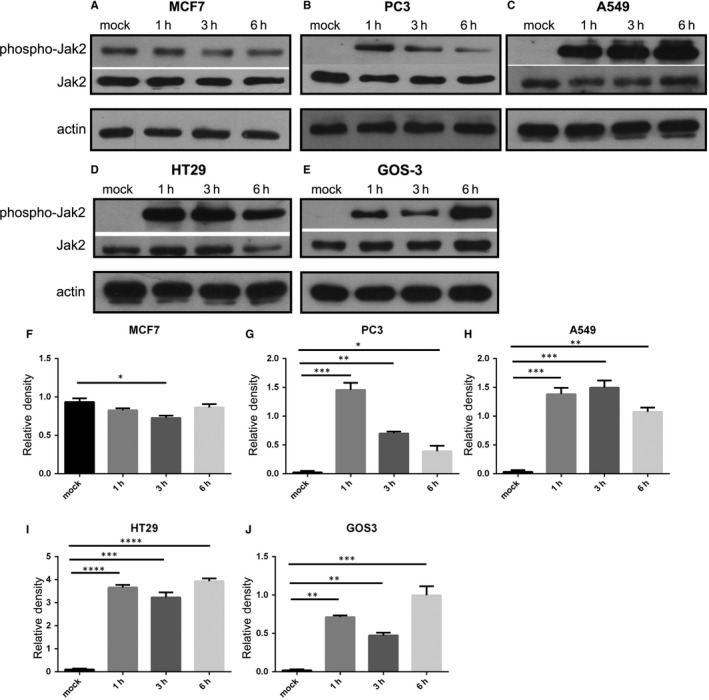
Effect of PC1 inhibition on the Jak pathway in cancer cell lines. (A‐E) Western blot analysis showing the effect of IgPKD1 on the phosphorylation of Jak2 in MCF7, PC3, A549, HT29 and GOS3 cells. (F‐J) Quantitative data showing the effect of IgPKD1 on the phosphorylation of Jak2 in MCF7, PC3, A549, HT29 and GOS3 cells. Bars represent means ± SD. *P < 0.05, **P < 0.01, ***P < 0.001, ****P < 0.0001 versus mock. Mock represents cells that have been incubated for 3 h with non‐immune rabbit serum; 1 h, 3 h, and 6 h represent time points of cell harvesting after 3 h incubation of cancer cells with the IgPKD1 antibody

Finally, we explored the effect of PC1 on the Jak pathway by treating cancer cells with IgPKD1 for 3 hours, followed by analysis of protein extracts at different time points (1, 3, and 6 hours). According to our results, the phosphorylation of Jak2 in PC3 (Figure 7B,G), A549 (Figure 7C,H), HT29 (Figure 7D,I) and GOS3 (Figure 7E,J) cells increased in IgPKD1‐treated cells compared to mock cells where Jak2 phosphorylation was negligible. On the other hand, the phosphorylation of Jak2 in MCF7 cells decreased in IgPKD1‐treated cells compared to mock cells (Figure 7A,F). All the above indicate that PC1 up‐regulates Jak signalling in MCF7 cells, while it down‐regulates Jak signalling in PC3, A549, HT29 and GOS3 cells. We also observed that this in vitro effect of PC1 on Jak2 phosphorylation status was time‐dependent. For example, in PC3 cells, the phosphorylation of Jak2 gradually decreased over time (1, 3 and 6 hours) in IgPKD1‐treated cells compared to mock cells (Figure 7B,G). These data further support that PC1 is linked in vitro to the Jak pathway in cancer cells.

## 4 DISCUSSION

Considering the common cellular features and signalling pathways between PKD and cancer, we speculated whether polycystins PC1 and PC2 play a role in cancer biology. First, we sought to evaluate the mRNA and protein levels of polycystins in cell lines from different types of cancer, including glioblastoma, prostate, lung, breast and CRC. We detected both mRNA and protein of PC1 and PC2 in the cancer cell lines. Our results also showed that there were differences in PC1 and PC2 protein levels between cancer cells (PC3, GOS3, HT29, MCF7) and their respective normal cells (HPrEc, CHLA‐259, CACO2, MCF10A). The protein expression of polycystins and their subcellular localization have been studied primarily in renal tissues and cultured cell lines of renal epithelial origin. With respect to our study, PC1 and PC2 protein expression has been detected in normal developing brain, breast ductal epithelium, colonic epithelium, prostate epithelium and lung epithelium.^48^, ^49^, ^50^ Nevertheless, immunohistochemistry data on polycystin protein expression in human tissues have not been consistent. Although in the present study we evaluated the protein expression of PC1 and PC2 in cancer and normal cell lines, it is also important to determine the subcellular localization of both polycystins because it is essential to their function. The subcellular localization of polycystins is complex and still debated by researchers. Results based on renal epithelial cells demonstrate that PC1 and PC2 are found on the primary cilia and in other subcellular compartments and membrane domains. Their localization, particularly PC2, has been found to be regulated by chemical chaperones, proteasome inhibitors, protein‐protein interactions and phosphorylation. In addition, given that PC1 and PC2 physically interact via their cytoplasmic C‐terminal tails, it is possible that they modulate each other's subcellular localization, but this remains controversial.^51^ Similarly to renal epithelial cells, all cell lines used in our study, apart from glioblastoma cells (GOS3), are epithelial in origin. However, whether PC1 and PC2 localization in our cancer epithelial cell lines follows the same pattern as in normal renal epithelial cells needs to be investigated in future studies.

For our next experiments, we focused on PC1 because of its large size, flexible nature, participation in cell‐cell and cell‐matrix contacts and known communication with many downstream signalling pathways via its intracellular C‐terminal tail.^7^, ^8^, ^9^, ^10^, ^11^, ^12^, ^13^, ^14^, ^15^ First, we evaluated the effect of PC1 on two important cellular features, cell proliferation and migration, which are commonly deregulated in cancer.^28^ Increased cell proliferation is a major feature of a polycystic kidney; cysts have even been characterized as ‘neoplasia in disguise’.^52^ Several studies have reported that PC1 also regulates cell migration.^53^, ^54^, ^55^, ^56^, ^57^ We found that blocking PC1 in vitro with IgPKD1 affected both cell proliferation and migration in cancer cells in a cell type‐dependent manner. According to our results, PC1 functions as a tumour‐suppressor protein in A549 cells inhibiting cell migration. In contrast, PC1 probably acts as an oncogene protein in GOS3 cells enhancing cell migration. Since PC1 has been reported to promote cell migration, could it be that GOS3 glioblastoma cells hijack this function of PC1 and turn it into a malignant signal that enhances their migratory and invasive abilities? Concerning cell proliferation, our results show that PC1 might be a tumour‐suppressor protein in MCF7, A549 and HT29 cells that impedes cell proliferation. Conversely, in GOS3 cells PC1 appears to be an oncogene that promotes cell proliferation. Because PC1 has been shown to inhibit cell proliferation in non‐cancerous cells,^12^, ^39^, ^51^, ^58^ we wondered whether MCF7, A549 and HT29 cancer cells are deregulated in such a way that interferes with the normal PC1‐mediated inhibition of proliferation. Do cancer cells achieve this effect by abolishing the function of the PC1 protein to transmit inhibitory signals to the cell's interior or by making the targets of these signals insensitive to inhibition? More study is required to confirm the influence of PC1 on cancer cell proliferation and migration and to uncover the mechanisms of this effect.

Next, we wanted to determine if PC1 regulates signalling pathways that are constitutively activated in cancer. Cancer and PKD are frequently accompanied by aberrant activation of the mTOR pathway.^29^, ^30^, ^31^, ^32^, ^33^, ^34^ Previous data have demonstrated that PC1 overexpression in SW480 colon cancer cells leads to down‐regulation of mTOR signalling.^43^ Jak signalling also becomes up‐regulated in cancer^35^, ^36^, ^37^ and studies have shown that PC1 activates Jak signalling in PKD.^38^, ^39^, ^40^, ^41^, ^42^ Therefore, we investigated if the mTOR and Jak pathways are affected by changes in the function of PC1 in cancer cells. Inhibiting PC1 via the use of IgPKD1 resulted in alterations in the phosphorylation level of upstream regulators and downstream effectors of mTOR and Jak2. Likewise, silencing PC1 gene expression via siRNA modified the phosphorylation level of mTOR pathway‐associated molecules and Jak2. Specifically, p‐p70 in PC3 cells, p‐mTOR in HT29 cells and p‐Jak2 in GOS3 cells were up‐regulated in both assays, and p‐p70 in GOS3 cells was down‐regulated in both assays. These findings indicate that PC1 stimulates mTOR signalling and inhibits Jak signalling in GOS3 cells, while it suppresses mTOR signalling in PC3 and HT29 cells. The mTOR suppression observed in HT29 colon cancer cells is consistent with the previous finding that PC1 down‐regulates mTOR signalling in SW480 colon cancer cells.^43^ These results also suggest that the effect of PC1 on mTOR signalling in cancer is cell type‐dependent. Moreover, these data contribute to our knowledge on the regulation of mTOR and Jak signalling in cancer. It should be noted that the conclusions on whether the mTOR cascade is activated or inhibited in cancer cells after changes in PC1 function are based only on the phosphorylation of a single mTOR‐related protein. Likewise, we focused only on Jak2 phosphorylation and did not evaluate any downstream effectors or target genes as surrogate markers of Jak pathway activity. Therefore, our conclusions in terms of Jak signalling activation are solely based on the phosphorylation status of Jak2. The inhibitory or activating effect of PC1 on the two cascades has to be validated through further studies.

A challenge that we encountered in the present study was the following: the two methods used to inhibit PC1 activity, PC1 knockdown with siRNA and PC1 inhibition with IgPKD1, did not generate the same results for most cancer cell lines as far as Jak pathway activity is concerned. The siRNA experiment data propose that PC3 and A549 cells use PC1 in order to activate Jak signalling, while MCF7 and GOS3 cells use PC1 to suppress Jak signalling. In contrast, data from the antibody‐mediated PC1 inhibition experiment imply that PC3, A549, HT29 and GOS3 cells use PC1 to down‐regulate Jak signalling, whereas MCF7 cells use PC1 to up‐regulate Jak signalling. These discrepancies could be due to limitations inherent in the two methods used to perturb the function of PC1; in contrast to RNAi where the PC1 protein is absent, the IgPKD1‐inhibited PC1 protein may lack certain activities but may still execute other activities and/or interact with other proteins. Moreover, both methods can have substantial off target effects.

In summary, our study demonstrates that PC1 regulates cell proliferation and migration and interacts with mTOR and Jak signalling in various cancer cell lines. Given that there is a lack of prior research on the subject of polycystins and cancer biology, this study represents the first steps towards understanding the function of polycystins in the pathophysiology of cancer. As we expected, our research prompted more question than answers. Future research should focus on the mechanism through which PC1 promotes or inhibits cell proliferation and migration, and the molecular details of the interaction between PC1 and mTOR and Jak signalling. Moreover, future studies on polycystins and cancer should explore whether polycystins are associated with any other signalling pathways in cancer cells. It would also be interesting to evaluate the clinical relevance of polycystins in cancer by studying human cancer tissues. All the above will reveal the significance of polycystins in cancer biology and may lead to the identification of new therapeutic targets or prognostic markers in cancer.

## CONFLICT OF INTEREST

The authors declare that there is no conflict of interest.
