# Supplementary material for: Polycystin‐1 affects cancer cell behaviour and interacts with mTOR and Jak signalling pathways in cancer cell lines
Source: J Cell Mol Med. 2019 Jun 28;23(9):6215–27. doi: 10.1111/jcmm.14506 (PMC6714176; doi:10.1111/jcmm.14506)
Supplement: Supplementary file 1 [file JCMM-23-6215-s001.doc]

**Figure S1** Knockdown efficiency of the *Pkd1* siRNA in cancer cell lines confirmed by quantitative Real-Time PCR (qRT-PCR). A, MCF7 cancer cell line. B, PC3 cancer cell line. C, A549 cancer cell line. D, HT29 cancer cell line. E, GOS3 cancer cell line. siRNA-PKD1 represents cells transfected with siRNA targeting the mRNA of *Pkd1*; siRNA-nt represents cells transfected with non-targeting siRNA.
