# Supplementary figures and images for: Polycystin‐1 affects cancer cell behaviour and interacts with mTOR and Jak signalling pathways in cancer cell lines
Source: J Cell Mol Med. 2019 Jun 28;23(9):6215–27. doi: 10.1111/jcmm.14506 (PMC6714176; doi:10.1111/jcmm.14506)

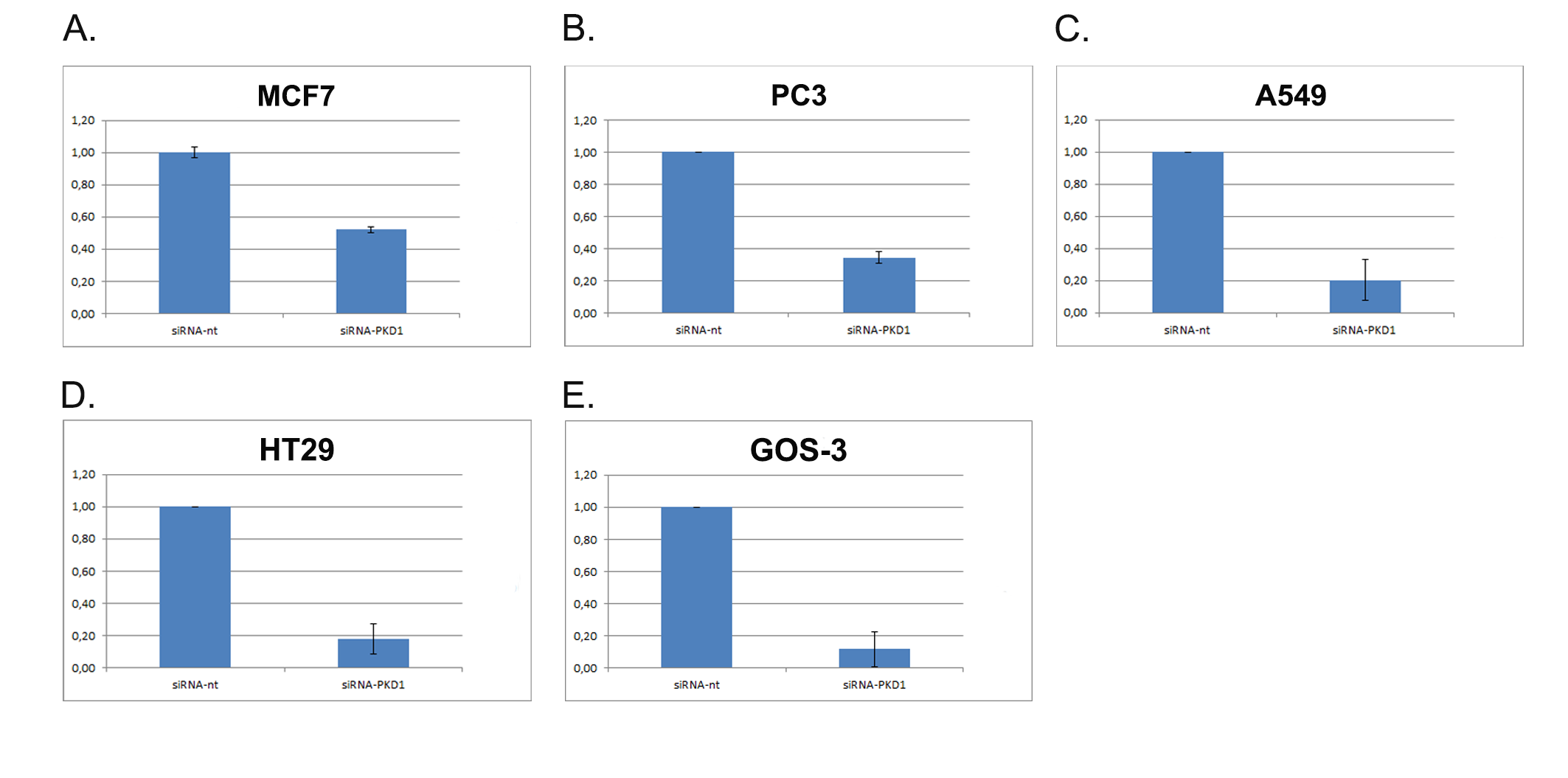

Supplement: Supplementary file 2 [file JCMM-23-6215-s002.tif]
